# Differential Effects of Low-Molecular-Weight Organic Acids on the Mobilization of Soil-Borne Arsenic and Trace Metals

**DOI:** 10.3390/toxics5030018

**Published:** 2017-08-21

**Authors:** Obinna Elijah Nworie, Junhao Qin, Chuxia Lin

**Affiliations:** 1 School of Environment and Life Science, University of Salford, Greater Manchester M5 4WT, UK; O.Nworie@edu.salford.ac.uk (O.E.N.); qinjunhao2015@gmail.com (J.Q.); 2 Key Laboratory of Agro-Environment in the Tropics, Ministry of Agriculture, South China Agricultural University, Guangzhou, 510642, China

**Keywords:** organic acid, arsenic, trace metal, mobilization, complexation, reductive dissolution

## Abstract

A batch experiment was conducted to examine the effects of six low-molecular-weight organic acids on the mobilization of arsenic and trace metals from a range of contaminated soils. The results showed that the organic acids behaved differently when reacting with soil-borne As and trace metals. Oxalic acid and acetic acid had the strongest and weakest capacity to mobilize the investigated elements, respectively. The solubilisation of iron oxides by the organic acids appears to play a critical role in mobilizing other trace metals and As. Apart from acidification and complexation, reductive dissolution played a dominant role in the dissolution of iron oxides in the presence of oxalic acid, while acidification tended to be more important for dissolving iron oxides in the presence of other organic acids. The unique capacity of oxalic acid to solubilize iron oxides tended to affect the mobilization of other elements in different ways. For Cu, Mn, and Zn, acidification-driven mobilization was likely to be dominant while complexation might play a major role in Pb mobilization. The formation of soluble Fe and Pb oxalate complexes could effectively prevent arsenate or arsenite from combining with these metals to form solid phases of Fe or Pb arsenate or arsenite.

## 1. Introduction

Environmental risk assessment and remediation of contaminated lands require knowledge of contaminant mobility. Currently, mobility evaluation for soil-borne contaminants relies on the extraction of their “mobile” or “mobilizable” fractions using selected chemical reagents that are hardly encountered in soils [1,2,3]. Consequently, the usefulness and reliability of such information for predicting contaminant mobility and bioavailability is less certain.

Low-molecular-weight organic acids (LMWOAs) are commonly present in soils as a result of root exudation and the microbially mediated decomposition of soil organic matter [4,5]. LMWOAs are capable of solubilizing trace metals and metalloids in the rhizosphere through soil acidification, complexation, and reduction reactions [6,7,8]. Except for highly acidic soils such as sulfidic soils where trace metals are largely mobilized by sulfuric acid [9,10], trace metals and metalloids tend to be tightly bound to the soil matrix in less and non-acidic soils [11,12,13]. Therefore, LMWOAs have a significant role to play in mobilizing soil-borne trace metals/metalloids, particularly in rhizospheric soils, which has important implications for the uptake of trace metals and metalloids by plants. In theory, LMWOAs should be superior to non-naturally occurring chemical reagents when being used as extracting agents for the evaluation of trace metal/metalloid mobility and bioavailability in contaminated soils.

Although there have been reports on the mobilization of trace metals/metalloids in soils by organic acids [14,15,16], there is still a large gap in knowledge about the chemical behavior of different trace metals and metalloids in the presence of different LMWOAs. Mixed results are often observed from different experiments regarding the effects of LMWOAs on liberation of soil-borne trace metals and metalloids. This is largely attributable to the complex nature of the reaction systems involving so many affecting factors. In addition, many of the investigations only focused on a limited number of either organic acids or trace metals/metalloids [17,18,19,20,21]. There is insufficient understanding of the differential effects of LMWOAs on the release of various trace metals and metalloids on a comparable basis. In this study, a range of soils with different levels of contamination by multiple trace metals and arsenic were used to compare the effects of six common LMWOAs, as well as an inorganic acid, on extracting trace metals and arsenic from the investigated soils. The objectives were to (a) assess the capacity of different organic acids to mobilize each of the investigated metals; (b) explain the observed differential effects; and (c) identify the major drawbacks of the metal extraction procedure using the inorganic acid as an extractant.

## 2. Materials and Methods

### 2.1. The Soil Samples Used in the Experiment

Twenty three soil samples were collected from a closed landfill site in the Greater Manchester region, United Kingdom. In the laboratory, the soil samples were oven-dried at 40 °C until they were completely dry. The samples were then ground to pass a 2 mm stainless steel sieve. Soil particles with a diameter >2 mm were discarded. The <2 mm soil fraction of each sample was homogenized and stored in an air-tight re-sealable polyethylene bag prior to use for analysis and the incubation experiment.

The total metal concentration of these samples ranged from 256 to 2469 mg/kg for As, from 44 to 180 mg/kg for Cu, from 27,957 to 59,013 mg/kg for Fe, from 14 to 777 mg/kg for Mn, from 260 to 1457 mg/kg for Pb, and from 25 to 150 mg/kg for Zn. Soil pH, electrical conductivity, and organic matter content had a range of 3.32–5.31, 0.027–0.250 dS/m, and 1.2–12%, respectively.

### 2.2. The Organic Acid Incubation Experiment

A batch experiment was conducted to observe the release of various trace metals and arsenic from the soil to the solution in the presence of organic acids. Six common LMW organic acids were used in this study, including acetic acid, citric acid, formic acid, malic acid, oxalic acid, and tartaric acid. The concentration of all the six organic acids was set at 200 mmol/L. 125 mL plastic bottles were used as batch reactors. For each soil sample, 50 mL of a relevant organic acid solution was added into the plastic bottle containing 10 g of the soil sample. Immediately after the solution addition, the bottle was hand-shaken for 1 min and then allowed to stand for 7 days. All the bottles were randomly placed in a covered paper box to avoid exposure to light during the entire period of the incubation experiment. At the end of the experiment, each bottle was hand-shaken for 1 min and then 15 mL of supernatant was taken, put into a 15 mL polystyrene centrifuge tube, and centrifuged by a MSE Mistral 1000 Centrifuge at a speed of 3600 rotations per minute (rpm) for 15 min. Centrifugation was required in order to remove suspended materials in the solution samples prior to the analysis of metals and arsenic. After centrifugation, each solution sample was transferred into a clean polystyrene tube and stored in a fridge at 4 °C before being analysed.

### 2.3. The Extraction of Metals by 1 M HNO_3_

The acid-mobilizable pool of metals was also evaluated using the conventional inorganic acid extraction method. For each soil sample, 50 mL of 1 M HNO_3_ was added into a 125 mL plastic bottle containing 10 g of soil sample. The bottles were shaken in a rotary shaker for 1 h at room temperature. After shaking, 15 mL of supernatant was taken, put into a 15 mL polystyrene centrifuge tube, and centrifuged by a MSE Mistral 1000 Centrifuge at a speed of 3600 rpm for 15 min. Centrifugation was required in order to remove suspended materials in the solution samples prior to the analysis of the metals. After centrifugation, each solution sample was transferred into a clean polystyrene tube by passing through a 0.45 µm membrane filter and was stored in a fridge at 4 °C before being analysed.

### 2.4. Analytical Methods

For the initial soil characterization, pH and the electrical conductivity of the soil samples were measured in a 1:5 (soil:water) extract using a calibrated Mettler Toledo 320 pH meter and a Mettler Toledo electrical conductivity meter, respectively. Total metal concentration was determined using a Niton XL2 Gold Hand-held XRF Analyzer. The instrument was calibrated by firstly analysing the 73,308 standard reference materials prior to sample analysis. To ensure the accuracy and reliability of the results obtained, all analyses were performed in duplicates and the analysis time was set at 240 s. Soil organic matter content was determined using a Walkley-Black method.

For the soil extraction and incubation experiment, trace metals and arsenic in the solution samples were measured using a Varian 720-ES inductively coupled plasma optical emission spectrometer (ICP-OES).

### 2.5. The Statistical Analysis Method

Statistical analysis of the experimental data was performed using IBM SPSS software Version 13.0.

## 3. Results

### 3.1. Arsenic and Trace Metals Released from the Soils by LMWOA

**Figure 1:**
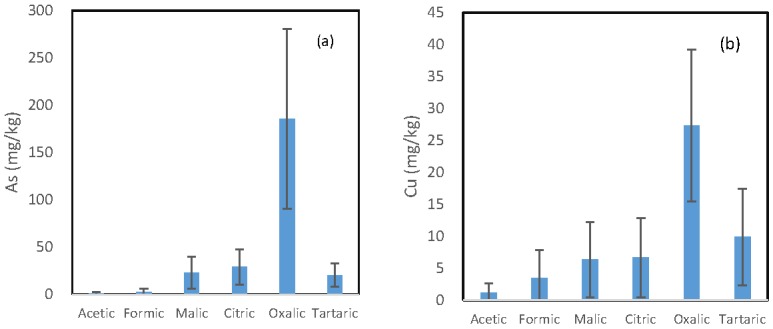

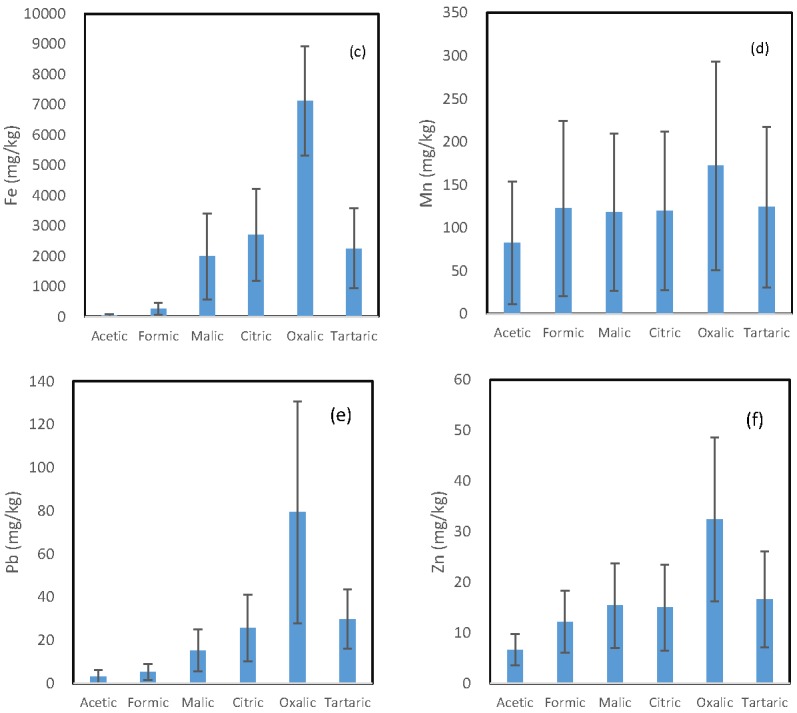
Mean concentration of (**a**) arsenic; (**b**) copper; (**c**) iron; (**d**) manganese; (**e**) lead; and (**f**) zinc being released from the soils by the six organic acids.

The mean concentration of each trace element released by the six LMWOAs is shown in Figure 1. A similar pattern is observed for the six investigated trace elements, and the oxalic acid incubation solutions and the acetic acid incubation solutions had the highest and the lowest concentration of each element, respectively. However, the difference in the concentration of each element between the oxalic acid-extractable form and other organic acid-extractable forms varied from element to element.

The ratio of the oxalic acid-extractable form to the second highest organic acid-extractable form was in the following decreasing order: As (6.38) > Cu (2.70) > Fe (2.63) = Pb (2.63) > Zn > (1.88) > Mn (1.39). Malic, citric, and tartaric acid incubation solutions had comparable concentration for all the six investigated elements. For Mn and Zn, their concentrations in the formic acid solution were also comparable to those in the above three solutions.

### 3.2. The Relationship between Different Organic Acid-Extractable Metal Forms

There was no relationship in As between the oxalic acid-extractable form and any of the other organic acid-extractable forms. However, for the other five organic acids (acetic, formic, malic, citric, and tartaric acids), there was a close relationship in the extractable As for any pairs of these organic acids. For Cu, Mn, and Zn, there was a close relationship in each of these three metals between any pair of the six organic acid-extractable forms. Similar to As, there was no relationship in Fe or Pb between the oxalic acid-extractable form and any other organic acid-extractable forms. Acetic Fe was only related to tartaric Fe but closely related to formic Fe. Formic Fe, citric Fe, and tartaric Fe were all interrelated to each other. Acetic Pb was not related to any Pb forms extracted by other organic acids (Table 1).

### 3.3. The Mobility of As and Its Relationship with Mobilizable Fe

**Figure 2:**
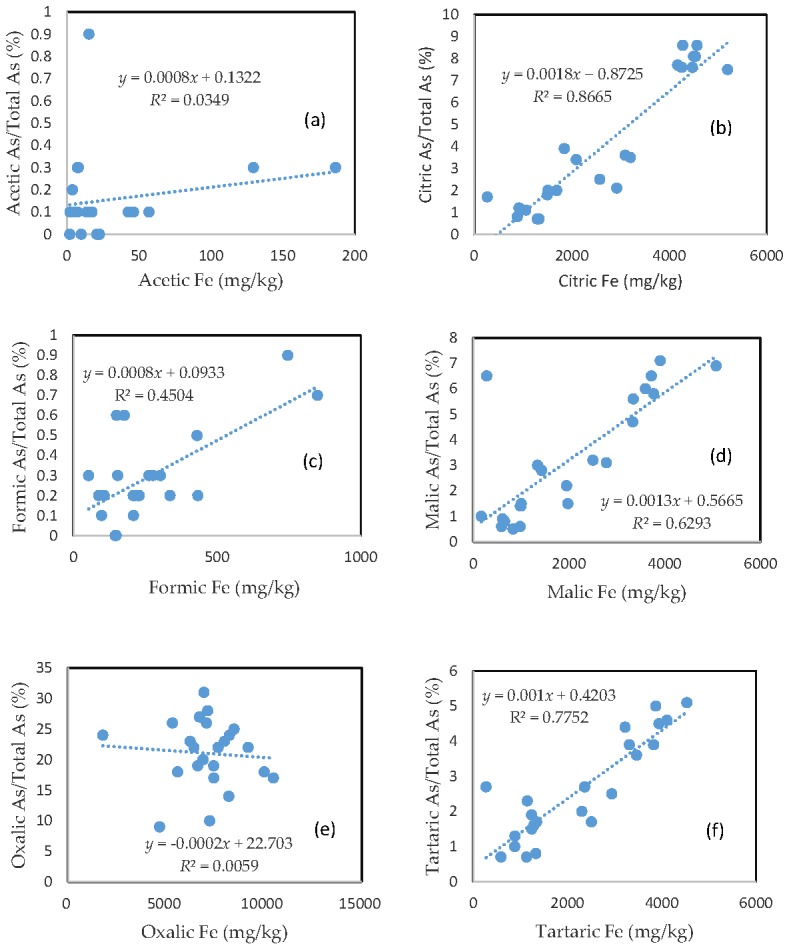
Relationship between the amount of Fe released from the soil and the rate of As mobilization in (**a**) acetic acid; (**b**) citric acid; (**c**) formic acid; (**d**) malic acid; (**e**) oxalic acid; and (**f**) tartaric acid treatments.

The percentage of organic acid-extractable As in the total As contained in the soils (i.e., the fraction of As mobilization) for each organic acid is plotted against the corresponding organic acid-extractable Fe fraction (Figure 2). Less than 1% of the total soil As was mobilized by either acetic acid or formic acid (Figure 2a,c). In the citric, malic, and tartaric acid treatments, the rate of As mobilization was less than 10% of the total soil As (Figure 2b,d,f). Oxalic acid treatment led to the release of about 10–30% of the total As from the soils (Figure 2e). There was a close relationship between the rate of As mobilization and the amount of Fe released from the soils for citric, malic, and tartaric acid treatments. A good relationship between the rate of As mobilization and the amount of Fe released from the soils was also observed for formic acid treatment. However, there was no clear relationship between the rate of As mobilization and the amount of released Fe for the acetic acid and oxalic acid treatments.

### 3.4. The Mobility of Cu and Its Relationship with Mobilizable Fe

**Figure 3:**
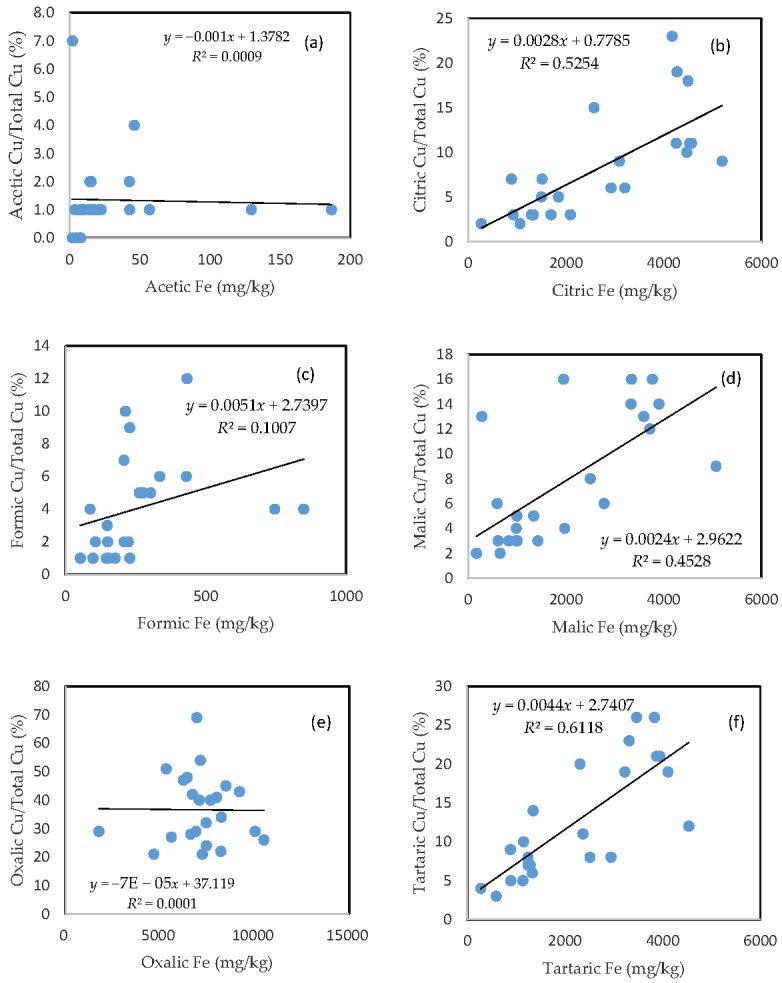
Relationship between the amount of Fe released from the soil and the rate of Cu mobilization in (**a**) acetic acid; (**b**) citric acid; (**c**) formic acid; (**d**) malic acid; (**e**) oxalic acid; and (**f**) tartaric acid treatments.

In comparison with As, the rate of Cu mobilization was much higher between 1 and 2%, between 3 and 20%, between 1 and 7%, between 2 and 16%, between 20 and 55%, and between 3 and 25% of the total Cu being released from the soils in the acetic, citric, formic, malic, oxalic, and tartaric acid treatments, respectively (Figure 3).

Similar to As, there was a good relationship between the rate of Cu mobilization and the amount of Fe released from the soils in the citric, malic, and tartaric acid treatments; a slight relationship between the rate of Cu mobilization and the amount of Fe released from the soils was also observed for formic acid treatment; and there was no clear relationship between the rate of Cu mobilization and the amount of released Fe for acetic and oxalic acid treatments.

### 3.5. The Mobility of Pb and Its Relationship with Mobilizable Fe

**Figure 4:**
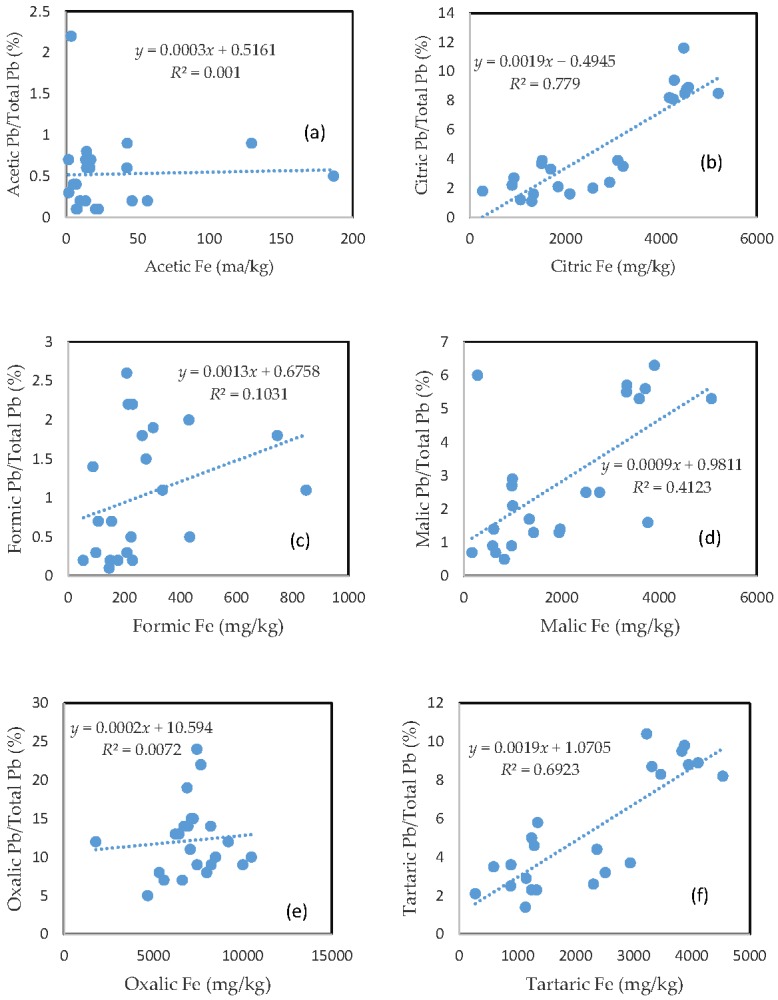
Relationship between the amount of Fe released from the soil and the rate of Pb mobilization in (**a**) acetic acid; (**b**) citric acid; (**c**) formic acid; (**d**) malic acid; (**e**) oxalic acid; and (**f**) tartaric acid treatments.

Very similar to Cu, the rate of Pb mobilization was closely related to the amount of Fe released from the soils for the citric acid, malic acid, and tartaric acid treatments (Figure 4b,d,f), and was slightly related to the amount of released Fe for formic acid treatment (Figure 4c). There was no clear relationship between the rate of Pb mobilization and the amount of released Fe for acetic and oxalic acid treatments (Figure 4a,e). The rate of Pb mobilization was less than 1%, 2.5%, and 6% for most of the samples in the acetic, formic, and malic acid treatments, respectively (Figure 4a,c,d).

Citric and tartaric acid treatments had a mobilization rate of between 1 and 10% of the total Pb for most of the samples (Figure 4b,f). About 5 to 25% of the total soil Pb was mobilized in the oxalic acid treatment (Figure 4e).

### 3.6. The Mobility of Zn and Its Relationship with Mobilizable Fe

**Figure 5:**
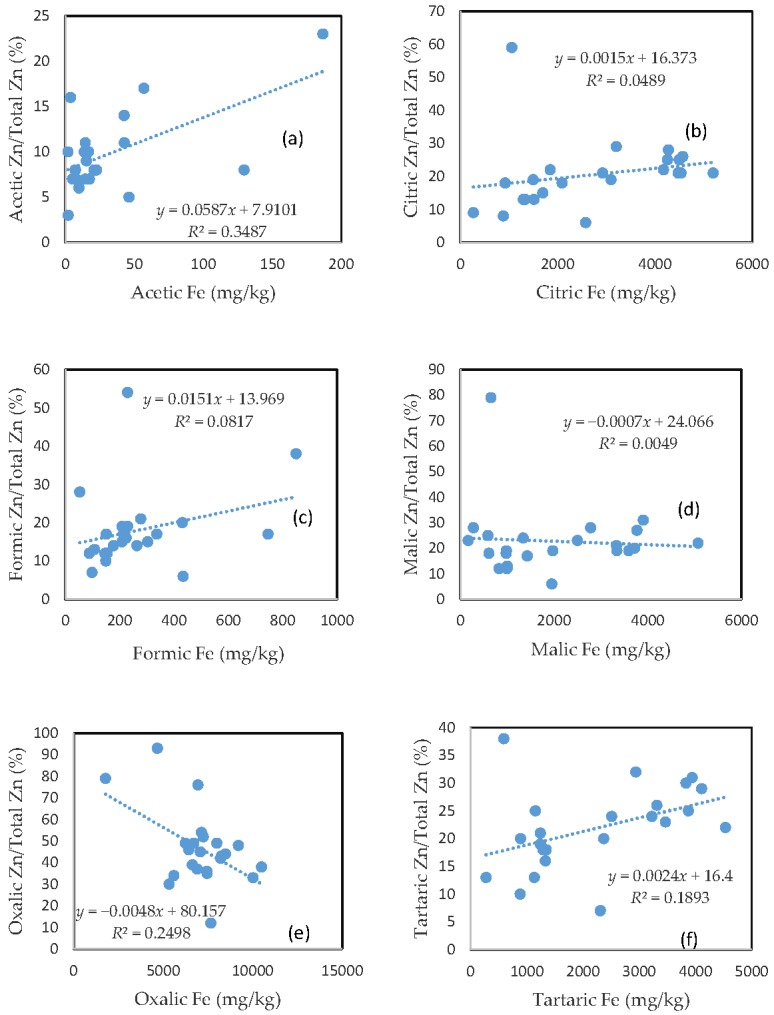
Relationship between the amount of Fe released from the soil and the rate of Zn mobilization in (**a**) acetic acid; (**b**) citric acid; (**c**) formic acid; (**d**) malic acid; (**e**) oxalic acid; and (**f**) tartaric acid treatments.

Largely unlike As, Cu, and Pb, there was no clear relationship between the rate of Zn mobilization and the amount of Fe released from the soils for the citric and malic acid treatments (Figure 5b,d). The tartaric acid-extractable Zn percentage was only slightly related to the amount of Fe released from the soils (Figure 5f).

On the other hand, the acetic acid-extractable Zn percentage showed a relationship with the amount of released Fe (Figure 5a), and the oxalic acid-extractable Zn percentage was even negatively related to oxalic acid-extractable Fe (Figure 5e). The poor relationship between the Zn extractable percentage and extractable Fe for the formic acid treatment was consistent with those for As, Cu, and Pb. In general, there was a markedly higher rate of Zn mobilization, as compared to other investigated elements, especially for the acetic and formic acid treatments.

### 3.7. The Relationship between HNO_3_-Extractable Fraction and Oxalic Acid-Extractable Fraction

**Figure 6:**
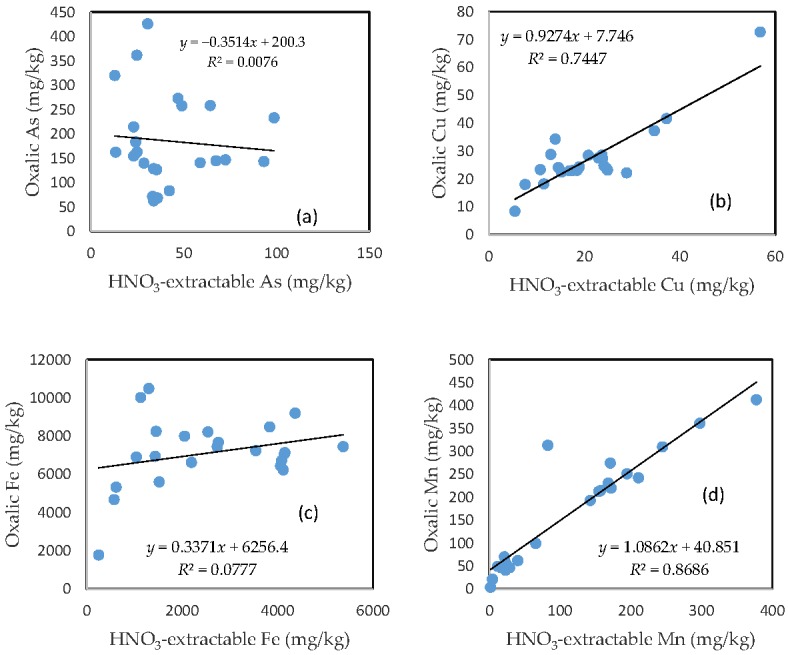

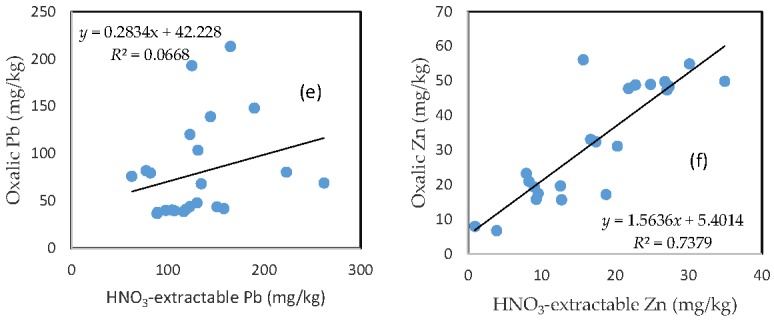
Relationship between the HNO_3_-extractable fraction and the oxalic acid-extractable fraction for (**a**) As; (**b**) Cu; (**c**) Fe; (**d**) Mn; (**e**) Pb; and (**f**) Zn.

There was no clear relationship between the HNO_3_-extractable form and the oxalic form for As, Fe, and Pb (Figure 6a,c,e). In contrast, HNO_3_-extractable Cu, Mn, and Zn were closely related to oxalic Cu, Mn, and Zn, respectively (Figure 6b,d,f). The slope for HNO_3_-extractable Cu vs. oxalic Cu and HNO_3_-extractable Mn vs. oxalic Mn is close to 1 (Figure 6b,d), while the slope of the HNO_3_-extractable Zn vs. oxalic Zn is more than 1.5 (Figure 6f).

### 3.8. The Relationship between the HNO_3_-Extractable Fraction and the Citric Acid-Extractable Fraction

**Figure 7:**
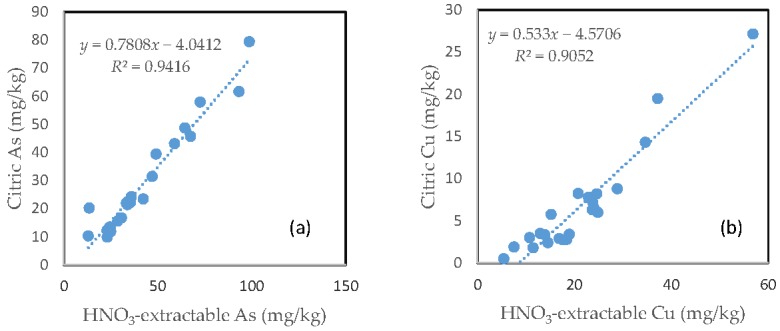

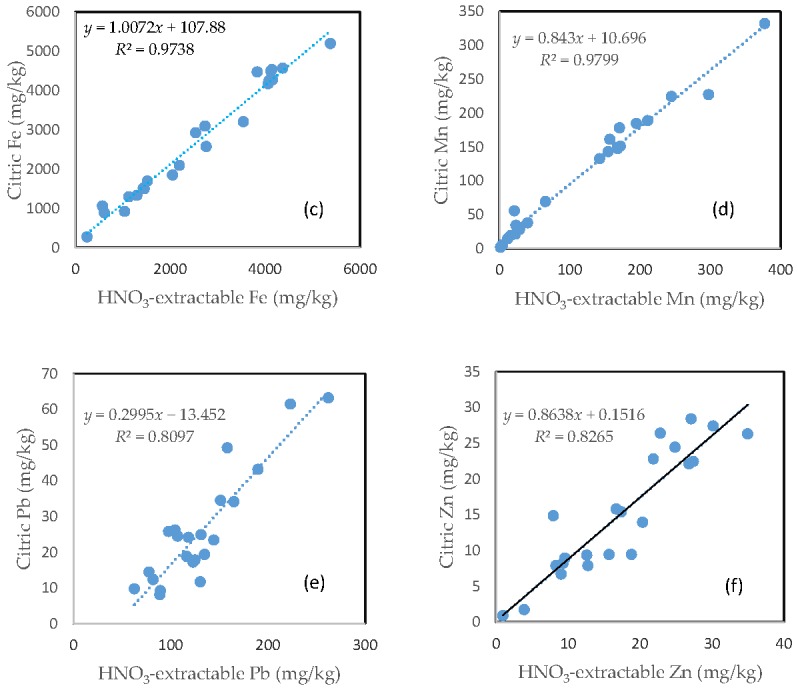
Relationship between the HNO_3_-extractable fraction and the citric acid-extractable fraction for (**a**) As; (**b**) Cu; (**c**) Fe; (**d**) Mn; (**e**) Pb; and (**f**) Zn.

In contrast with the oxalic acid treatment, there was a very close relationship between the HNO_3_-extractable fraction and the citric acid extractable fraction for all the six investigated trace metals. However, the slope of regression line differed among the different elements. Fe had a slope of nearly 1, the slope dropped to below 0.9 for Zn, Mn and As. The slope for Cu was only about 0.5 and Pb only had a slope of less than 0.3. The regression equation showed a negative intercept for As, Cu and Pb (Figure 7).

### 3.9. The Relationship between the HNO_3_-Extractable Fraction and the Malic Acid-Extractable Fraction

**Figure 8:**
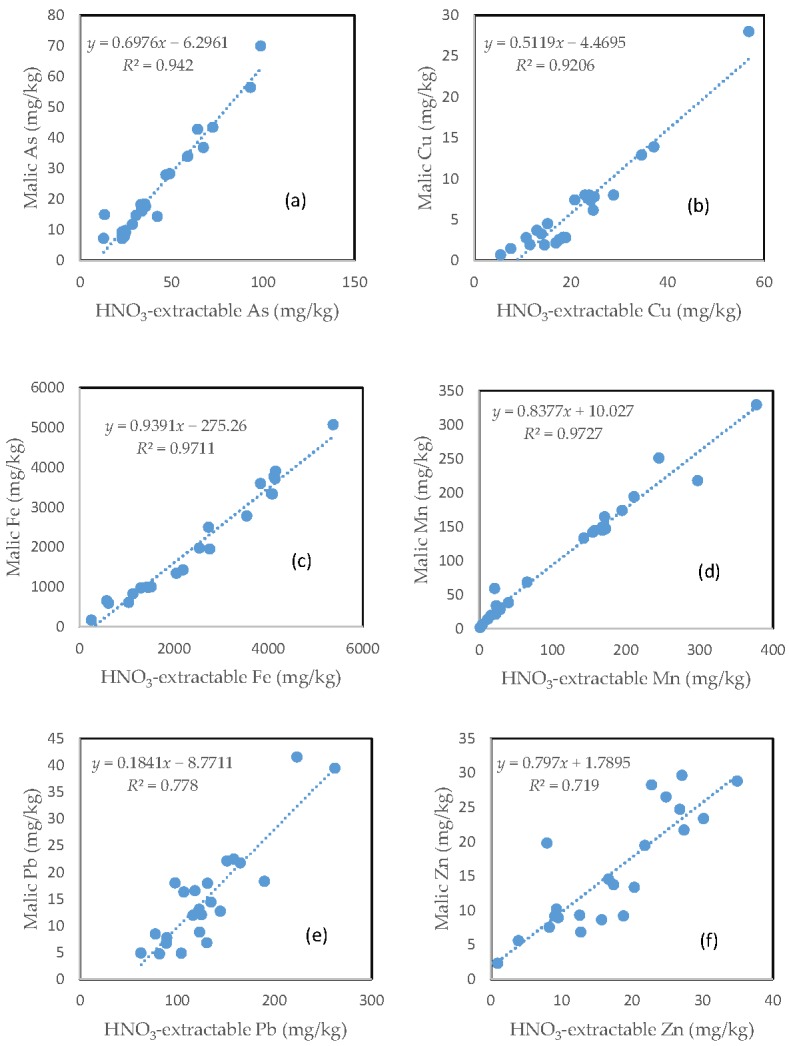
Relationship between the HNO_3_-extractable fraction and the malic acid-extractable fraction for (**a**) As; (**b**) Cu; (**c**) Fe; (**d**) Mn; (**e**) Pb; and (**f**) Zn.

Largely similar to the citric acid treatment, there was a very close relationship between the HNO_3_-extractable fraction and the malic acid extractable fraction for all the six investigated trace elements (Figure 8). However, the slope of regression line differed among the different elements. Fe had the highest slope (0.9391), followed by Mn (0.8377), Zn (0.7970), As (0.6976), and Cu (0.5119). Pb only had a slope of less than 0.2. The regression equation showed a negative intercept for As, Cu, and Pb (Figure 8a,b,e).

## 4. Discussion

The poor relationship between the oxalic acid-extractable Fe and the extractable Fe by other organic acids (Table 1) suggests that the dominant mechanism responsible for mobilization of Fe in the oxalic acid treatment was significantly different from those in other organic acid treatments. Dissolution of iron oxides by organic acids involves the initial adsorption of organic ligands on the iron oxide surface and the subsequent release of iron ions from the solid surfaces through either non-reductive or reductive dissolution pathways [22]. The stronger capacity of oxalic acid, relative to some other organic acids, to solubilize iron oxides was previously observed and attributed to the high affinity of the oxalate species to the iron oxide surfaces [23] and oxalic acid’s high acid strength, good complexing capacity, and reducing power [24]. The results obtained from this study are generally consistent with these findings. While the similar pattern observed for manganese may be, to some extent, due to the same reasons [25], the markedly reduced gap in extractable Mn between oxalic acid and other organic acids suggests that oxalic acid did not work much better than non-oxalic acids in terms of mobilizing soil-borne Mn. This may be due to the fact that manganese oxides are more resistant to oxalic acid-driven reduction, as compared to iron oxides. It is interesting to note that oxalic acid-extractable Mn was closely related to some of the non-oxalic acid-extractable Mn, suggesting that Mn tended to behave similarly in the presence of either oxalic acid or other organic acids in this study. Probably under the set experimental conditions, Mn mobilization was primarily driven by acidification and complexation rather than reductive dissolution (when the soil-borne Mn reacts with oxalic acid).

Oxides of iron and manganese play important roles in binding arsenic and trace metals in soils [26,27,28,29]. Consequently, the high degree of similarity in the pattern of organic acid-extractable fractions observed for As, Cu, Pb, and Zn (Figure 1) may indicate that the release of these elements are, to a certain extent, associated with the dissolution of iron and manganese oxides in the presence of organic acids. Like Fe, oxalic acid-extractable As and Pb were not related to the non-oxalic acid-extractable As and Pb, respectively. This appears to suggest that As and Pb in the oxalic acid solution also behaved differently from those in other organic acid solutions. Oxalic acid was likely to mobilize certain As and Pb species that could not be easily solubilized by other organic acids.

The close relationship between the rate of As mobilization and the amount of Fe released from the soils for the citric, malic, and tartaric acid treatments suggests that these acids effectively corroded the surfaces of the iron oxide particles, leading to the mobilization of As and the trace metals that were bound to the mineral surfaces. In the case of oxalic acid treatment, the much stronger corrosion of iron oxides led to the liberation of substantial amounts of iron from the deeper layers of iron oxide particles that contained fewer trace metals and As. This may explain the poor relationship between the rate of As mobilization and the amount of Fe released from the soils in the oxalic acid treatment. The close relationship among citric, formic, malic, and tartaric acid-extractable fractions for each element (Table 1) suggests that these organic acids behaved similarly in terms of solubilizing the trace metals and As in the soils.

The close relationship between the HNO_3_-extractable fraction and the oxalic acid-extractable fraction for Cu, Mn, and Zn further confirms that the mobilization of these three metals in the oxalic acid treatment was predominantly driven by acidification, while the poor relationship between the HNO_3_-extractable fraction and the oxalic acid-extractable fraction for As, Fe, and Pb points to other mechanisms. The formation of Fe and Pb oxalate complexes, which is not necessarily dependent on pH condition, was likely to be the dominant process that kept these metals in the solution. This could also prevent the combination of arsenate or arsenite with Fe and Pb to form precipitates [30,31].

In contrast with oxalic acid treatment, there was a close relationship between the HNO_3_-extractable fraction and either the citric acid-extractable fraction or the malic acid-extractable fraction for all the six investigated elements. This suggests that for these two organic acids, the major driving force for mobilizing As and trace metals was similar to that for HNO_3,_ i.e., acidification.

The research findings in this study have implications for evaluating the mobility of As and trace metals in contaminated soils. While the traditional inorganic acid extraction procedure may, to certain degree of reliability, allow estimation of mobilizable metal pools in the presence of many LMW organic acids, it fails to give any sensible indication of the oxalic acid-mobilizable pools of As and trace metals. Since different types of organic acids have different effects on mobilizing As and trace metals in soils. The composition of organic acids in rihzospheric soils is therefore very important in terms of the controls on the dynamics of trace metals and metalloids in soil-plant systems.

The results also provide information that can be used for developing soil remediation techniques such as soil washing and phytoextraction. Clearly oxalic acid is much more effective and efficient for enhancing the mobility and phytoavailability of soil-borne As and trace metals.

## 5. Conclusions

Under the set experimental conditions, LMWOAs behaved differently when reacting with soil-borne trace metals and As. Oxalic acid and acetic acid had the strongest and weakest capacity to mobilize the six investigated elements, respectively. Malic, citric, and tartaric acids showed a similar element-mobilizing capacity and formic acid had the capacity somewhere between this group of acids and acetic acid for most of the elements. The differential effects of various LMWOAs on element mobilization also varied from element to element with Mn and Zn showing smaller differences in mobility among the six LMWOAs, as compared to As and other trace metals.

It was likely that solubilisation of iron oxides by the LMWOAs played a critical role in mobilizing other trace metals and As. The driving force for the dissolution of iron oxides in the presence of oxalic acid was markedly different from that in the presence of other LMWOAs. Apart from acidification and complexation, reductive dissolution played a dominant role in the dissolution of iron oxides in the presence of oxalic acid, while acidification tended to be more important for dissolving iron oxides in the presence of other LMWOAs.

The unique capacity of oxalic acid to solubilize iron oxides tended to affect the mobilization of other elements in different ways. For Cu, Mn, and Zn, acidification-driven mobilization was likely to be dominant, while complexation might play a major role in Pb mobilization. The formation of soluble Fe and Pb oxalate complexes could effectively prevent arsenate or arsenite from combining with these metals to form solid phases of Fe or Pb arsenate or arsenite.

## Figures and Tables

**Table 1:** Correlation matrices of six datasets involving various organic acid-extractable metals (1) As, (2) Cu, (3) Fe, (4) Mn, (5) Pb, and (6) Zn.

	**Acetic As**	**Formic As**	**Malic As**	**Citric As**	**Oxalic As**	**Tartaric As**
Acetic As	1					
Formic As	0.851**	1				
Malic As	0.639**	0.743**	1			
Citric As	0.612**	0.696**	0.991*	1		
Oxalic As	0.052	0.071	-0.016	-0.059	1	
Tartaric As	0.666**	0.740**	0.936**	0.925**	0.229	1
						
	**Acetic Cu**	**Formic Cu**	**Malic Cu**	**Citric Cu**	**Oxalic Cu**	**Tartaric Cu**
Acetic Cu	1					
Formic Cu	0.928**	1				
Malic Cu	0.852**	0.975**	1			
Citric Cu	0.813**	0.950**	0.972**	1		
Oxalic Cu	0.876**	0.932**	0.909**	0.890**	1	
Tartaric Cu	0.821**	0.956**	0.989**	0.981**	0.903**	1
						
	**Acetic Fe**	**Formic Fe**	**Malic Fe**	**Citric Fe**	**Oxalic Fe**	**Tartaric Fe**
Acetic Fe	1					
Formic Fe	0.912**	1				
Malic Fe	0.385	0.495*	1			
Citric Fe	0.362	0.536**	0.848**	1		
Oxalic Fe	0.189	0.204	0.107	0.267	1	
Tartaric Fe	0.421*	0.579**	0.816**	0.980**	0.285	1
						
	**Acetic Mn**	**Formic Mn**	**Malic Mn**	**Citric Mn**	**Oxalic Mn**	**Tartaric Mn**
Acetic Mn	1					
Formic Mn	0.964**	1				
Malic Mn	0.958**	0.989**	1			
Citric Mn	0.962**	0.994**	0.996**	1		
Oxalic Mn	0.935**	0.985**	0.983**	0.989**	1	
Tartaric Mn	0.949**	0.987**	0.984**	0.991**	0.996**	1
						
	**Acetic Pb**	**Formic Pb**	**Malic Pb**	**Citric Pb**	**Oxalic Pb**	**Tartaric Pb**
Acetic Pb	1					
Formic Pb	0.213	1				
Malic Pb	0.213	0.905**	1			
Citric Pb	0.247	0.894**	0.923**	1		
Oxalic Pb	0.114	0.153	0.092	0.114	1	
Tartaric Pb	0.263	0.841**	0.883**	0.946**	0.181	1
						
	**Acetic Zn**	**Formic Zn**	**Malic Zn**	**Citric Zn**	**Oxalic Zn**	**Tartaric Zn**
Acetic Zn	1					
Formic Zn	0.837**	1				
Malic Zn	0.767**	0.933**	1			
Citric Zn	0.836**	0.965**	0.966**	1		
Oxalic Zn	0.643**	0.839**	0.801**	0.868**	1	
Tartaric Zn	0.854**	0.948**	0.914**	0.979**	0.898**	1

** Correlation is significant at the 0.01 level (2-tailed); * Correlation is significant at the 0.05 level (2-tailed).
